# Obituary: Armando Felsani (1947–2022)

**DOI:** 10.1038/s41420-022-01208-w

**Published:** 2022-10-07

**Authors:** Marco G. Paggi

**Affiliations:** IRCCS - Regina Elena National Cancer Institute, Via Elio Chianesi 53, 00144 Rome, Italy

**Keywords:** Cell division, Cancer

Armando Felsani passed away on 21 April 2022. He has been a pioneer in investigating myogenesis and myogenic differentiation, a field in which he produced seminal work regarding the interplay between *MyoD*, the master gene of muscle differentiation, *RB1*, the retinoblastoma tumour suppressor gene, and epigenetic factors in muscle differentiation, and how oncoproteins (including of viral origin) can disrupt these processes. Such research interests later brought him to delve into cancer cell cycle regulation by tumour suppressors, cyclins and cyclin-dependent kinases in cancer onset and progression.
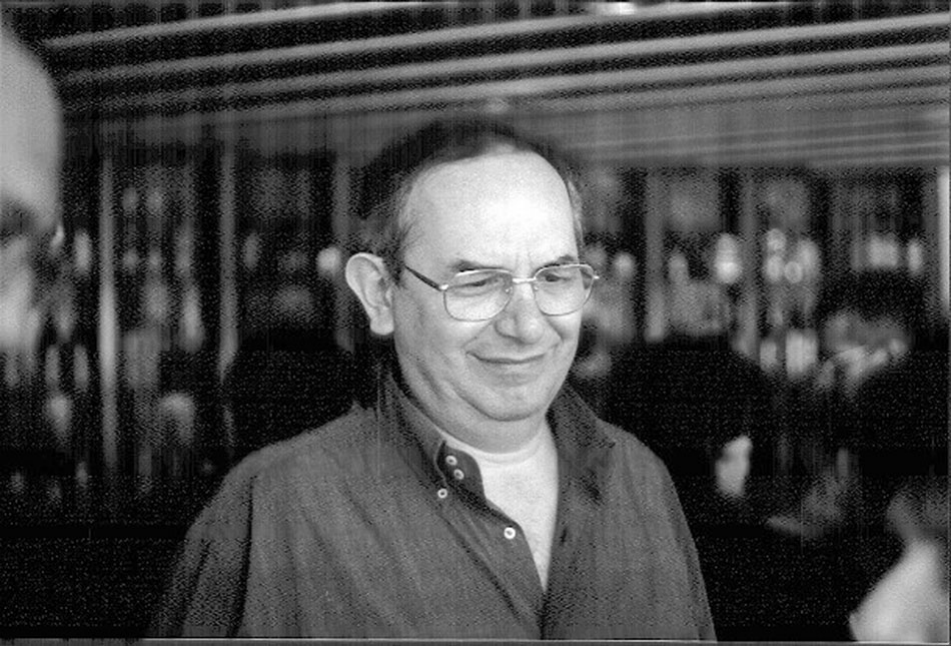


Armando was born in 1947 in Rome, Italy, where he graduated in Biology at University La Sapienza in 1971. His scientific career began at the Institute of Molecular Embryology of the National Research Council (CNR) in Naples, an Institute that fostered then excellent research in molecular biology in Italy. There, Armando began to study how RNA populations, and particularly polysomal RNA, which was then regarded as the functionally relevant expression of the genome, varied during development and differentiation in various model systems. That interest in the molecular bases of cell differentiation were to accompany all his research career. He then moved to England and later to France, where he worked with François Gros as an EMBO long-term fellow at the Collège de France in Paris: there, his interest in differentiation took a sharper focus on the neurogenic and myogenic pathways. On returning to Italy, in 1978, he joined the Biopathology Department of University La Sapienza in Rome, where a large group, then led by Pablo Amati, was studying how oncogenic viruses “hijack” and disrupt master factors of differentiation. It was there that Armando started his long career as a CNR scientist, which he has pursued with ever renewed enthusiasm and search for innovation until retirement.

Anyone who has got to know Armando could not avoid being attracted by his culture, personal kindness and—above all—a passionate willingness to advance scientific knowledge. This was coupled with an extraordinary collaborative and problem-solving attitude: that combination determined an immense commitment to solve all those problems, both conceptual and practical, that any researcher must face every day. For anyone in his institution and beyond, he was a reference for identifying and applying correct experimental methodologies and was a tireless advocate for the importance of data reproducibility. Not surprisingly, Armando has been an unsurpassed tutor for a vast number of young scientists.

I met Armando in 1990, when he and his group moved to the Regina Elena National Cancer Institute in Rome, my current workplace. I approached him while searching for a mentor to refine my approach towards science and, as an added value, I found a true friend. Fortunately, our scientific partnership went on for many years, in which our common interest in tumour suppressor genes and proteins led us to collaborate on the hot topic of cancer cell cycle and the molecular mechanisms underlying the ability of Human Papillomavirus and Adenovirus oncoproteins to disable cell growth suppressor factors.

As over the years Armando had become a true enthusiast and profound connoisseur not only of the technologies underlying complex “omics” platforms used in biomedical research, but also of every novelty in hardware and software in the broader sense, he decisively contributed to the birth and growth of researchers’ “technological personality” and ability to interface and coexist in harmony with informatics and bioinformatics, in the lab as well as in the everyday life. For example, at the dawn of the World Wide Web, Armando provided the knowledge necessary to connect our research institute to the global scientific community. In those days, we had the tools, but neither the culture nor the knowledge to face the upcoming Internet revolution. That was back in 1991, when even navigating databases and using the earliest available analysis software was difficult and “unfriendly” to many.

The synergy between his ability to study, think, conceive top level experimental work both in life sciences as well as in informatics has allowed all those who have been close to him, and the institutions in which he worked, to make the quantum leap necessary to behave as leaders in a world in continuous technological evolution.

One of Armando’s strengths was to never back down from challenging experimental complexities that scientists of his generation have witnessed, from the birth of genome cloning and sequencing era. Indeed, after his retirement from the CNR, he continued his scientific activity at Genomnia, a Milan-based pioneer company in Next Generation Sequencing and bioinformatics analysis. Here, his passion for molecular biology and computer sciences, combined with renewed energy, leading him to accept the challenge and give unique contributions to set up new protocols and address a variety of experimental queries.

Beyond his remarkable scientific skills, Armando possessed immense human qualities that made the interaction with him an extremely pleasant experience, concerning any topic, from DNA sequencing to jazz. He has been a truly beloved husband, father and grandfather.

Armando will be remembered and sorely missed.

